# Body-size distributions and size-spectra: universal indicators of ecological status?

**DOI:** 10.1098/rsbl.2010.0240

**Published:** 2010-05-05

**Authors:** Owen L. Petchey, Andrea Belgrano

**Affiliations:** 1 Department of Animal and Plant Sciences, University of Sheffield, Sheffield, UK; 2 Swedish Board of Fisheries, Institute of Marine Research, Lysekil, Sweden

**Keywords:** allometry, taxonomy, ecosystem assessment

## Abstract

The sizes of individual organisms, rather than their taxonomy, are used to inform management and conservation in some aquatic ecosystems. The European Science Foundation Research Network, SIZEMIC, facilitates integration of such approaches with the more taxonomic approaches used in terrestrial ecology. During its 4-year tenure, the Network is bringing together researchers from disciplines including theorists, empiricists, government employees, and practitioners, via a series of meetings, working groups and research visits. The research conducted suggests that organismal size, with a generous helping of taxonomy, provides the most probable route to universal indicators of ecological status.

## 1. Introduction

In 1953, Wallace H. Coulter was granted a patent for a ‘Means of counting particles suspended in a fluid’, and the technology described gave rise to the Coulter counter, a machine that could count and measure the size of particles, including small organisms. When a sample of water was run through the machine, particles with a diameter from less than 1 µm to about 100 µm were counted (Sheldon *et al*. 1972). One could then ask how many individual plankton cells were recorded in particular size ranges, say 1–2 µm, 2–4 µm, 4–8 µm, and so on. The resulting relationships between abundance and size were dubbed size spectra, and they revealed remarkable regularities in pelagic community structure (Kerr & Dickie 2001).

Treating organisms as particles differing only in size is an often controversial viewpoint, not well integrated with taxonomy-focused research. Coupling these two viewpoints is the overarching goal of research being coordinated by the European Science Foundation SIZEMIC Research Network, lead by Richard Law (University of York, UK) and Julia Blanchard (Imperial College, London, UK). Its main aims are to: (i) integrate size-based and species-based ecological research; (ii) provide a focus and mechanism for initiating and strengthening collaborations across existing research boundaries (e.g. ecosystem boundaries); and (iii) create training opportunities for young scientists. Three working groups are supported by SIZEMIC: (i) human impacts on food webs—are there patterns across ecosystems, and can taxonomic and size based approaches be integrated? (led by Frank Van Veen, University of Exeter, Cornwall Campus, UK); (ii) testing the generality of Elton's rule: comparing aquatic and terrestrial ecosystems across environmental conditions (led by Julia Reiss, Queen Mary, University of London, UK); and (iii) body size and redundancy: across system comparisons (led by Ute Jacob, Alfred Wegner Institute, Bremerhaven, and Owen Petchey, University of Sheffield, UK). The remainder of this Meeting Report highlights some of the background research, advances and opportunities associated with the SIZEMIC Research Network.

## 2. Patterns and theory of size spectra

**Figure 1.**
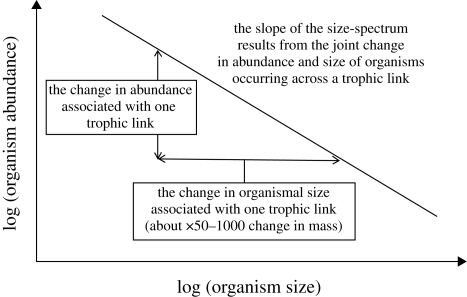
Size spectra describe the relationship between organism size and abundance and can be predicted from the expected joint change in abundance and organismal mass that occurs across one trophic link. The theory behind the scaling of abundance and mass is extensive and includes important nuances. These and other distributions and relationships of size and mass, their inter-relationships, mathematical derivations and estimation methods are described in a number of detailed publications and their appendices (Brown & Gillooly 2003; Andersen & Beyer 2006; White *et al*. 2007; Reuman *et al*. 2008).

Analyses of the slopes of size spectra are now widely used to assess the state of marine ecosystems at regional and global scales (Shin *et al*. 2005). Observed size spectra typically become steeper (more negative) following exploitation (mainly of fishes); in one survey of fishes the slope of the size spectra became about 1.5 times steeper over the period from 1977 to 1993 (Rice & Gislason 1996). Demonstrating detectable effects of exploitation on size spectra has been key to their emergence as indicators of marine ecosystems (figure 1).

A rich body of theory exists for predicting the slope of size spectra, and this theory can be used to calculate reference states in fisheries (Jennings & Blanchard 2004). Jennings and Blanchard found that achieving a slope as steep as that observed in the North Sea requires an unfeasibly low predator∶prey mass ratio (of around 10) and/or trophic transfer efficiency (around 0.0025). This suggests that the North Sea is a long way from the theoretical unexploited reference state. This potential for size spectra to provide indicators of ecosystem status, and to allow estimates of distance from reference state, has probably contributed to their use as general indicators of marine ecosystem status (Shin *et al*. 2005).

Are size spectra, and other local allometries, less useful in non-marine ecosystems? Are fishes and the ecosystems they inhabit so different from other species and ecosystems that a universal approach is unsuitable and inapplicable? Is the direct exploitation of larger species, which in part causes the steeper size spectra, so different from other environmental impacts, such as altered nutrient levels, habitat destruction and species invasions?

The answer to these questions appears to be ‘no’: perhaps somewhat surprisingly, size spectra theory even appears to apply in some soil ecosystems. Christian Mulder and his colleagues studied 12 managed grasslands and 10 ex-organic farms abandoned for at least a decade (Mulder & Elser 2009). Differences in management practices resulted in soil ecosystems differing greatly in soil pH and nutrient ratios. Soils were sampled for the abundance and mass of bacteria, fungi, nematodes, mites, springtails and enchytraeids (e.g. earthworms), and size spectra constructed. Low pH and relatively high ratios of phosphorus to carbon and nitrogen were associated with steeper size spectra, resulting from the relative rarity of larger organisms and abundance of smaller organisms in high phosphorus soils. These links between soil chemistry, farming practices and the characteristics of size spectra indicate the possibility of assessing the status of soil ecosystems, even across large geographical ranges and soil types that are very difficult to compare using more traditional taxonomic indicators. Detailed analysis and modelling of some of the soil biodiversity data collected by Mulder's group, and of one estuarine and two pelagic communities, revealed broad agreement between the theory and observations (Reuman *et al*. 2008). It seems that the systematic changes in size spectra that occur in exploited fisheries are occurring in other systems under other types of environmental pressure.

## 3. Differences among ecosystems

While there may be general perception that terrestrial and aquatic ecosystems differ fundamentally, there have been relatively few systematic and quantitative analyses of the size-structuring of communities across ecosystem types, while controlling as carefully as possible for differences in types of feeding and organism. Making thorough quantitative comparison of the size structure of communities is one of the focal questions addressed by the SIZEMIC network. For example, how does the relationship between a species' body mass and trophic level depend on ecosystem type? Does the dependence of interaction strength on body size vary across ecosystem types? To what extent do findings depend on the taxonomic range of the organisms considered? These are important questions if we are to gauge and understand the general importance of body size for species interactions and community structure.

Another critical issue that could continue to cloud studies of the size involves the question ‘The size of what?’ In Brose *et al*.'s (2006) extensive empirical study of predator∶prey mass ratios, species had an average body size, and interactions occurred between species. In reality, individuals interact (not species), and these individuals have sizes often quite different from the species' average. Few studies record individuals interacting; fewer record information about individuals, such as size (Ings *et al*. 2009). Those that do reveal how different individual- and species-based analyses of size structure can be. One such study assembled gut contents of more than 4000 individuals from a freshwater stream (Woodward & Hildrew 2001). Each individual or fragment of an individual was measured and converted to individual mass. Aggregating to the species level made some consumers appear to feed on resources nearly 100 times larger than themselves (Woodward & Warren 2007). However, the same data indicated that individual predators never had in their gut a prey individual larger than themselves. Clearly, the effects of aggregation up to the species level can have large and potentially misleading effects on our perception of the size structure of communities.

## 4. Coupling size and taxonomy

Important progress will be achieved if size-based views are reconciled and merged with ones more focused on taxonomy and species identity. This is already happening, for example, by incorporating aspects of species' taxonomic identities into fisheries models that previously differentiated individuals only by their size (Andersen & Beyer 2006). While the growth of fishes is indeterminate, different species of fishes grow to particular (asymptotic) sizes. Andersen and Beyer's model predicts that the abundance of species is related to asymptotic size, while also predicting the individual-based size spectra. Another example (Blanchard *et al*. 2009) comes from creation of a model of an ecosystem containing organisms with a variety of feeding characteristics, some feeding according to size (the predators) and some feeding on shared unstructured resources largely according to taxonomy (the detritivores). In this model, the slope of the resulting size spectra depended on the strength of coupling between the two components. Such theory, which adds components of taxonomic identity to otherwise entirely size-structured models complements research by adding information about body size and its consequences to methods that were previously almost entirely taxonomic. The majority of food web research has focused on networks of taxonomic entities linked (or not) by trophic interactions. Adding information about species body sizes can explain variation in the structure of taxonomic food webs (Cohen *et al*. 2003) and provide foraging-based explanations of the occurrence of feeding links and fluxes (Brose *et al*. 2008; Petchey *et al*. 2008).

**Figure 2.**
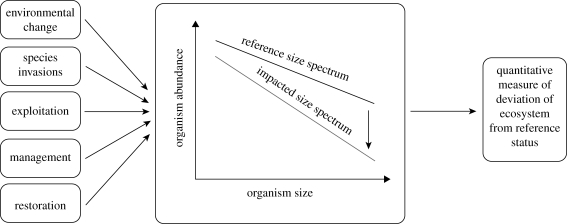
Universal size? The size of individuals in an ecological community is affected by many kinds of processes, from human exploitation to species extinctions. Ecological theory can predict the reference size spectrum. The European Science Foundation funded SIZEMIC Research Network is researching the potential for size spectra to incorporate elements of taxonomy to produce universal indicators of ecosystem status.

Ultimately, ecologists will benefit from a clearer understanding of the joint importance of taxonomy and size, how their relative importance changes across ecosystems, and the mechanisms responsible for differences in relative importance. Such information would go a long way to providing a simultaneous understanding of both the generalities (e.g. allometries) and the specifics of ecology that can be related also to biodiversity and biogeochemical flux studies in aquatic and terrestrial systems (Belgrano *et al*. 2002). It would also help answer the question in the title more definitively than is presently possible. The importance and universality of size, emphasized by links between size and physiological rates (West *et al*. 1997; Gillooly *et al*. 2001), provides the opportunity for it to become a first principle of assessment, management, and conservation of ecosystem status based on formal mathematical theory (e.g. figure 2). However, while body size and species identity are clearly critically important, we neither claim nor imagine that the development of a Coulter counter that could accommodate organisms of all sizes and taxonomies would necessarily be a magic bullet for ecosystem assessment. Realizing the full potential of size-based approaches to ecosystem, conservation certainly will require more research to find efficient, elegant, and general methods for including taxonomy.
